# Separation of Short-Chain Fatty Acids from Primary Sludge into a Particle-Free Permeate by Coupling Chamber Filter-Press and Cross-Flow Microfiltration: Optimization, Semi-Continuous Operation, and Evaluation

**DOI:** 10.3390/membranes15010022

**Published:** 2025-01-11

**Authors:** Nikhil Shylaja Prakash, Peter Maurer, Harald Horn, Florencia Saravia, Andrea Hille-Reichel

**Affiliations:** 1 DVGW-Research Center at the Engler-Bunte-Institute, Water Chemistry and Water Technology, Karlsruhe Institute of Technology, Engler-Bunte-Ring 9, 76131 Karlsruhe, Germany; harald.horn@kit.edu (H.H.); saravia@dvgw-ebi.de (F.S.); 2 Sewage Treatment Plant for Research and Education, Institute for Sanitary Engineering, Water Quality and Solid Waste Management, University of Stuttgart, Bandtäle 1, 70569 Stuttgart, Germany; peter.maurer@iswa.uni-stuttgart.de; 3 Engler-Bunte-Institute, Water Chemistry and Water Technology, Karlsruhe Institute of Technology, Engler-Bunte-Ring 9, 76131 Karlsruhe, Germany

**Keywords:** primary sludge, dark fermentation, short-chain fatty acids, flocculation, microfiltration, biorefinery

## Abstract

Short-chain fatty acids (SCFAs) are valuable metabolic intermediates that are produced during dark fermentation of sludge, which, when capitalized on, can be used as chemical precursors for biotechnological applications. However, high concentrations of solids with SCFAs in hydrolyzed sludge can be highly detrimental to downstream recovery processes. This pilot-scale study addresses this limitation and explores the recovery of SCFAs from primary sludge into a particle-free permeate through a combination of chamber filter-press (material: polyester; mesh size: 100 µm) and cross-flow microfiltration (material: α-Al_2_O_3_; pore size: 0.2 µm; cross-flow velocity: 3 m∙s^−1^; pressure = 2.2 bars). Firstly, primary sludge underwent dark fermentation yielding a hydrolyzate with a significant concentration of SCFAs along with total solids (TS) concentration in the range of 20 to 30 g∙L^−1^. The hydrolyzate was conditioned with hydroxypropyl trimethyl ammonium starch (HPAS), and then dewatered using a filter press, reducing TS by at least 60%, resulting in a filtrate with a suspended solids concentration ranging from 100 to 1300 mg∙L^−^^1^. Despite the lower suspended solids concentration, the microfiltration membrane underwent severe fouling due to HPAS’s electrostatic interaction. Two methods were optimized for microfiltration: (1) increased backwashing frequency to sustain a permeate flux of 20 L∙m^−^^2^∙h^−^^1^ (LMH), and (2) surface charge modification to maintain the flux between 70 and 80 LMH. With backwashing, microfiltration can filter around 900 L∙m_eff_^−2^ (without chemical cleaning), with the flux between 50 and 60 LMH under semi-continuous operation. Evaluating the particle-free permeate obtained from the treatment chain, around 4 gC_SCFAs_∙capita^−1^∙d^−1^ can be recovered from primary sludge with a purity of 0.85 to 0.97 C_SCFAs_∙DOC^−1^.

## 1. Introduction

Out of the influent load of 40 g_TOC_∙capita^−1^∙d^−1^, a sludge load of 24 g_TOC_∙capita^−1^∙d^−1^ is produced in a conventional municipal wastewater treatment plant (WWTP) [1]. Although sludge produced serves as an excellent source for biogas production, within the context of a wastewater biorefinery (WWBr), organic carbon in sludge can be tapped more efficiently to produce bio-based products that have more value in the economy in comparison with biogas [2]. Some of the bio-based products that have garnered increasing interest in recent years include bioplastics [3] and biohydrogen [4], typically due to their green nature. Therefore, research in the last decades has focused more towards the fermentation of short-chain fatty acids (SCFAs) from waste streams as they can serve as valuable precursors for biotechnological applications [5].

To operate an economically viable WWBr, the fermentation of SCFAs from the solids stream needs to be optimized, and dark fermentation is an effective method to produce SCFAs [6]. However, the amount of total solids converted to gas is very low during dark fermentation, and the solids are still mostly present in the suspended and dissolved phase. Hence, the total solids concentration between sludge and hydrolyzed primary sludge or hydrolyzate is similar. This means that the concentration of particles present in the hydrolyzate is very high and cannot be directly processed in biotechnological applications. It is also important to consider the diverse microflora present in sludge [7], which can interfere with downstream recovery processes. For instance, in their pilot-scale study on polyhydroxyalkanoate (PHA) accumulation using mixed cultures from industrial wastewater [8], the authors clearly outlined the importance of having a suspended solids-free substrate for better PHA yields. The authors also highlighted the impedance on PHA yields caused by organisms that are incapable of storing PHAs. Similarly, another pilot-scale study described that higher PHA yields could be achieved with particle-free wastewater [9]. In case of biohydrogen production using microbial electrolysis cells, the growth of methanogens has shown to compromise the overall hydrogen recovery process [10,11]. These drawbacks due to particles or microorganisms in sludge can be tackled by implementing membrane technology to produce a particle-free permeate for biotechnological use.

Microfiltration can offer an economical and technical advantage, as low-molecular-weight compounds can be separated from the particulate waste stream through a size sieving mechanism [12]. Ceramic membranes in particular have attracted increasing interest because of their narrow pore size distribution, robustness, wide range of pH tolerance, high porosity, and hydrophilicity, which leads to higher permeate fluxes and reduced fouling in comparison with polymeric membranes [13,14]. Indeed, the pore size is a crucial factor as it is important to allow only the dissolved compounds to pass through. Typically, the size range of microfiltration membranes can vary between 0.1 and 10 µm [15]. A membrane cut-off of 0.2 µm was found to be optimum [16], as the particles can be separated from SCFAs. Nevertheless, the authors demonstrated that a microfiltration membrane can reject up to 15% of SCFAs (within a cut-off range of 0.1 to 0.8 µm) due to adsorption on particles. Similarly, it was reported that dissolved organic compounds could be retained by a 0.2 µm ceramic membrane up to 20% due to the presence of high suspended solids concentrations [15]. Therefore, in this study, a chamber filter press was introduced as a pre-treatment stage to sieve off larger particles and reduce the suspended solids load on microfiltration.

In this study, experiments were carried out at pilot scale to treat primary sludge in a real WWTP. Dark fermentation was subjected to long-term experimentation under different organic loading rates, which could produce hydrolyzate with varying total solids concentration. The hydrolyzate was conditioned with a cationic flocculant, and then dewatered using a chamber filter press, which would then undergo cross-flow microfiltration. For this research, a starch-based flocculant was used due to its economic viability, environmental friendliness, and biodegradability in comparison with its synthetic counterpart, which has a hazardous nature [17]. In general, SCFAs produced in dark fermentation can generate a higher revenue than biogas when used for bio-based product recovery (like PHAs) [2]. However, the yields calculated for such bio-based products solely based on the performance of dark fermentation may not be practical, as the presence of particles can significantly impede the functioning of downstream processes. Hence, the novel treatment chain presented here at the pilot scale to produce a particle-free SCFA permeate via membrane technology offers a practical solution to the aforementioned issues due to particles, and could be a potential concept to sustain a functional WWBr. To the best of our knowledge, a combination of solids separation and dark fermentation to produce a substrate free of particles containing predominantly SCFAs has not been reported in the literature so far. The main objectives of this research were as follows: (1) sludge conditioning using a biodegradable cationic starch-based flocculant to produce low suspended solids filtrate for microfiltration, (2) identifying fouling and providing suitable remedies for long-term cross-flow microfiltration, and (3) evaluating the overall treatment chain in a semi-continuous mode for SCFA recovery at the pilot scale in terms of load and revenue generated.

## 2. Materials and Methods

### 2.1. Experimental Setup and Overview

**Figure 1:**
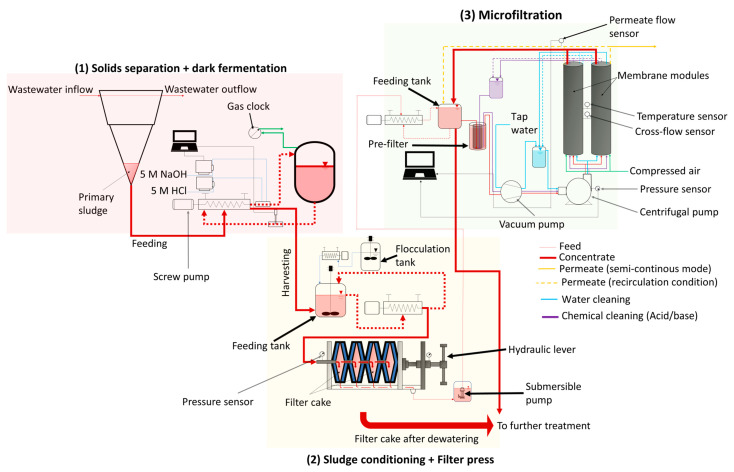
Schematic representation of the cascade comprising (1) solids separation and dark fermentation, (2) sludge conditioning and dewatering using a chamber filter press, and (3) microfiltration.

In this study, experiments were carried out at the pilot scale in a wastewater research facility (Buesnau, Germany). Based on the influent flowrate (24 m^3^∙d^−1^) into the primary sedimentation tank, and total suspended solids concentration (TSS) in the range of 50 to 500 mg∙L^−1^ in the inflow to this WWTP [18], around 100 to 150 L of primary sludge was produced per day. There are three major steps in this study: (1) dark fermentation of primary sludge to produce SCFAs, (2) conditioning of primary sludge after dark fermentation (i.e., hydrolyzate) with a starch-based flocculant followed by dewatering using a chamber filter press to reduce solids concentration, and (3) cross-flow microfiltration to recover SCFAs in a particle-free permeate. The three steps were optimized separately, and then were combined together and evaluated as a treatment chain. The descriptions of the experimental setup can be found in Section 2.1.1, Section 2.1.2, and Section 2.1.3. The schematic representation of the cascade can be found in Figure 1.

#### 2.1.1. Primary Sedimentation Tank Coupled with a Dark Fermentation Reactor

A primary sedimentation tank with a volume of 3 m^3^ was coupled with a dark fermentation reactor (made of stainless steel insulated with polyurethane), with a total volume of 0.3 m^3^. The working volume of the reactor was kept at 0.2 m^3^ (130 L of primary sludge was produced on average per day) to maintain a hydraulic retention time (HRT) of 1.5 d (changes in HRT are described in Section 3.2.1). A screw pump (Netzsch pumps and systems, Selb, Germany) with a flow rate of 4300 L∙h^−1^ (22 recirculations per hour), was used for homogenization and to harvest the hydrolyzate and feed the primary sludge. The screw pump at this flow rate experienced energy losses, which was transformed to heat. The polyurethane insulation of the reactor vessel may have prevented this heat from dissipating, resulting in temperature containment within the reactor and an eventual temperature rise; thus, a temperature of 32 °C could be maintained without using an external thermostat. Two dosage pumps (Prominent, Heidelberg, Germany) were implemented in the recirculation line of the setup to control the pH value. The pH value was adjusted using 5 M-HCl and-NaOH solutions. A pH electrode (Greisinger, Regenstauf, Germany) was also installed in the recirculation line, and the data were monitored using data acquisition software (Volker Preyl, Stuttgart, Germany). The flow rate of the gas produced was recorded using a drum-type gas meter (Ritter, Bochum, Germany). Gas samples were collected regularly from the headspace of the reactor, and the composition of the gases was analyzed using a gas chromatograph coupled with a helium ionization detector (HP 6890 series, Palo Alto, CA, USA).

#### 2.1.2. Sludge Conditioning and Dewatering at Lab and Pilot Scale

Firstly, the hydrolyzate that was harvested from dark fermentation underwent lab-scale flocculation experiments to determine optimum dosages of flocculant, which could then be used for the pilot-scale filter press. Flocculation was performed using 0.4 L beakers, which were agitated using stainless steel stirrers. For lab-scale (Section 3.1.1) and pilot-scale (Section 3.1.2) tests, potato-based starch (Emsland KCG 750, Emsland group, Emlichheim, Germany) was used. The starch-based flocculant was grafted with 2,3-epoxypropyltrimethylammonium chloride. The end product was hydroxypropyl trimethyl ammonium starch (HPAS). Details of the starch-based flocculants can be found elsewhere [19]. Following the dosage of HPAS at lab scale, the hydrolyzate underwent rapid stirring at 150 rpm (rotations per minute) for 10 min (for colloidal destabilization), followed by slow stirring at 80 rpm for 10 min to ensure floc formation. The hydrolyzate was then poured on to the sieves, which were subjected to gravity-driven filtration. These filtrations were carried out using a polyester-based sieve (mesh size: 630 µm; RAI-TILLIERES SAS, France), and stainless steel sieves (mesh size: 100 µm).

For pilot-scale experiments (experimented as a cascade in Section 3.2), pea-based starch (HKF CleanTech AG, Rotkreuz, Switzerland) was utilized. The change in the type of HPAS from the lab scale was due to the discontinued supply of the previous starch-based flocculant. In any case, using a similar grafting procedure as described previously, HPAS was generated as the end product. A stainless steel stirrer was used for rapid stirring to destabilize the colloids. A screw pump was used for slow mixing to promote floc formation. The total duration of mixing (rapid and slow) was between 15 and 20 min. Based on the average volume of the daily harvested hydrolyzate (around 130 L), 4 chambers were required per dewatering experiment. The chambers were lined with polyester-based sieves (mesh size: 100 µm), and clamped at a pressure of 400 bars using a hydraulic lever. Once the chambers were filled completely, the pressure in the chambers could be increased up to 15 bars. Filtration was stopped when the flow rate was very low, after which chambers were opened to remove the highly concentrated filter cake. A submersible pump was used to transfer the filtrate to a feed tank prior to microfiltration.

#### 2.1.3. Pilot-Scale Microfiltration

A preconfigured cross-flow filtration setup incorporated with pumps and sensors obtained from Atec, Ulm, Germany was used to treat the filtrate generated from the chamber filter press. In this research, two tubular ceramic microfiltration membranes (Inopor, Veilsdorf, Germany) were integrated into the filtration setup. The following are the details of the ceramic microfiltration membranes: material: α-Al_2_O_3_, pore size = 0.2 µm, length = 1.2 m, number of channels = 7, channel diameter = 6 mm, effective filtration area = 0.316 m^2^. A pre-filter (Atec, Ulm, Germany) with a pore size of 60 µm was utilized prior to cross-flow microfiltration to prevent larger particles from entering the membrane module. A manometer, permeate flow sensor, cross-flow sensor, and temperature sensor were part of the filtration setup, as shown in Figure 1. A vacuum pump sucked the feed liquid into the system, while a centrifugal pump generated the required cross flow. The inlet pressure was fixed at 2.2 bars. Separate external tanks depending on the feed, clean water, or cleaning solutions (acid or base) were used for microfiltration (see Figure 1). Microfiltration was either experimented in a recirculation condition or a concentrating condition. In the recirculation condition, the concentrate and permeate were directed to the feed tank to maintain a constant feed composition, while, in the concentrating condition, only the concentrate was recirculated back into the feed tank, whereas the permeate was collected separately. The former was performed to optimize the microfiltration process, while the latter was performed to replicate a realistic condition. Flux determined with clean water for the ceramic membrane or clean water flux (J_CW_) was found to be 135 $L\cdot {m_{eff}}^{-2}\cdot h^{-1}\;or\;LMH$. To recover the flux, membrane cleaning after filtration with the filtrate was performed as follows:

(1) Filtration with clean water at 40 °C for 1 h at 3 m∙s^−1^.
(2) Filtration with an alkaline solution (Atec, Ulm, Germany; Product no.: Atec 2610) at pH 12 at 40 °C for 1 h at 3 m∙s^−1^.
(3) Filtration with clean water at 40 °C for 0.5 h at 3 m∙s^−1^.
(4) Filtration with an acidic solution (Atec, Ulm, Germany; Product no.: Atec 3027) at pH 2 at 40 °C for 1 h at 3 m∙s^−1^.
(5) Filtration with clean water at 40 °C for 0.5 h at 3 m∙s^−1^.
(6) Chemical cleaning is exclusive to this study due to the type of wastewater used, and a detailed description can be found in Section 3.1.2.

### 2.2. Analytical Methods

Total organic carbon (TOC) was measured according to DIN EN 13137 [20]. Total solids (TS) and volatile solids (VS) were measured using standard methods (DIN 38414) [21]. Total suspended solids (TSS) and volatile suspended solids (VSS) were determined according to [22]. To measure the dissolved parameters, samples were first centrifuged at 4000 rpm. Then, the supernatant was filtered off using a glass fiber membrane with a pore size of 1 µm, followed by a regenerated cellulose membrane with a pore size of 0.45 µm. Dissolved organic carbon (DOC) was measured in accordance with DIN EN 1484 [23]. In addition, lactic acid and SCFAs (acetic acid (HAc), propionic acid (HPr), iso-butyric acid (Hbu-iso), butyric acid (Hbu), and iso-valeric and valeric acid (HVa-iso and HVA) from the filtered samples were detected using ion chromatography (IC) (881 Compact Pro (Metrohm, Switzerland)). Lactic acid had a high instability in production and was found only in very low concentrations. Therefore, lactic acid was not included in the calculations. All the above-mentioned parameters were measured in duplicate. SCFAs were measured on a daily basis for pilot-scale dark fermentation. In case of dewatering (lab and pilot scale), samples were measured in the feed (at the start), concentrate, and filtrate (at the end of the run). In the case of the optimization of microfiltration, parameters were measured on a daily basis. For semi-continuous microfiltration, parameters were measured once in the feed (at the start of filtration) and twice in the concentrate and permeate, once when half the volume recovery was reached, and finally at the end of the filtration run. Dissolved oxygen concentration (GMH 5630, Greisinger, Regenstauf, Germany) and oxidation reduction potential (WTW, Multi 350i, Xylem, Rye Brook, NY, USA) were measured occasionally in the dark fermentation reactor to ensure anaerobic conditions, as the reactor underwent regular feeding. Dissolved oxygen concentration was approximately 0 mg∙L^−1^, and ORP was between minus 350 mV and minus 390 mV.

### 2.3. Data Interpretation

Since this research focuses on two key steps—dark fermentation and membrane filtration—the equations are classified below in separate sections.

#### 2.3.1. Fermentation

The yields of SCFAs in dark fermentation, $Y_{SCFAS}$, were calculated using the following equation:

(1) $$Y_{SCFAs}\left[\%\right]=\left\{\frac{c_{SCFAs,out}\left[gC\cdot L^{-1}\right]\times \frac{V_{f/h}\left[L\right]}{V_{W}\left[L\right]\times dt(d)}}{OLR\left[g_{TOC}\cdot L^{-1}\cdot d^{-1}\right]}\right\}\times 100$$

where $c_{SCFAs,out}$ represents the mass concentrations of SCFAs (as carbon equivalents) in the effluent of the dark fermentation reactor. $V_{W}$ is the working volume, $V_{f/h}$ is the volume fed or harvested per day, and dt is the feeding cycle, which is one day. OLR is the organic loading rate, expressed as g of total organic carbon (TOC) fed per liter and day. Primary sludge was hydrolyzed when it entered the dark fermentation reactor, but this was not implemented in the equation. Since hydrolysis in the sedimentation zone was inevitable, yields were evaluated in the hydrolyzate.

#### 2.3.2. Filtration

Permeate flux ($J_{std}$) for microfiltration was standardized to 25 °C, and calculated using the following equation:

(2) $$J_{std}\left[L\cdot {m_{eff}}^{-2}\cdot h^{-1};LMH\right]=\frac{Q_{permeate}\left[\frac{L}{h}\right]}{A_{eff\left[m^{2}\right]}\times f_{T}}$$

where $Q_{permeate}$ is the permeate flow rate, $A_{eff}$ is the effective filtration area of the membrane (0.316 m^2^), and $f_{T}$ is the temperature correction factor. $f_{T}$ was calculated using a calibration curve between clean water flow rate and temperature (see supplementary Figure S2).

Cross-flow velocity ($v_{CF}$) was calculated according to the equation given below:

(3) $$v_{CF}\left[m\cdot s^{-1}\right]=\frac{Q_{CF}\left[\frac{m^{3}}{s}\right]}{A_{CS\left[m^{2}\right]}}$$

where $Q_{CF}$ is the volumetric flow rate in the tubular membrane channels, and $A_{CS}$ is the cross-sectional area of the membrane channels.

The dosage of HPAS (D_HPAS_) in the hydrolyzate was calculated as follows:

(4) $$D_{HPAS}\left[{mg}_{HPAS}\cdot {g_{TS}}^{-1}\right]=\frac{m_{HPAS}\left[mg\right]}{V_{hyd}\left[L\right]\times TS\left[\frac{g}{L}\right]}$$

where m_HPAS_ is the amount of HPAS dosed, V_hyd_ is the volume of hydrolyzate, and TS is the total solids concentration in the hydrolyzate.

The volume recovery ($V_{rec})$ after microfiltration or filter press was calculated as follows:

(5) $$V_{rec}(\%)=\frac{V_{p,f}\left[L\right]}{V_{f\left[L\right]}}\times 100$$

where $V_{p,f}$ is the final volume in the permeate (for microfiltration)/filtrate (filter press) after filtration and $V_{f}$ is the initial volume in the feed prior to the start of filtration.

Retention (R_x_) of a certain parameter (represented as x) was calculated according to the following equation:

(6) $$R_{x}(\%)=\left\{\frac{C_{in,x}-C_{out,x}\left[mg\cdot L^{-1}\right]}{C_{in,x}\left[mg\cdot L^{-1}\right]}\right\}\times 100$$

where $C_{in,x}$ and $C_{out,x}$ are the concentrations of a certain parameter x in the influent and effluent of the corresponding membrane process (chamber filter press or microfiltration), respectively.

#### 2.3.3. Recovery of SCFAs: Dark Fermentation–Filter Press–Microfiltration Cascade

To calculate the recovery of SCFAs (${Rec}_{SCFAs}$) after filtration, Equation (1) was modified as shown below to include $V_{rec}$ after subsequent filtration steps:

(7) $${Rec}_{SCFAs}\left[\%\right]=\left\{\frac{c_{SCFAs,f/p}\left[gC\cdot L^{-1}\right]\times V_{p,f}(L)}{OLR\left[g_{TOC}\cdot L^{-1}\cdot d^{-1}\right]\times HRT\left(d\right)\times V_{f/h}\left[L\right]}\right\}\times 100$$

where $c_{SCFAs,f/p}$ represents the mass concentrations of SCFAs (as carbon equivalents) in the filtrate or permeate. HRT is the hydraulic retention time.

The permeate quality after microfiltration (f_DOC_) was assessed based on the ratio of SCFAs (as carbon equivalents) to DOC:

(8) $$f_{DOC}\left[\%\right]=\left\{\frac{c_{SCFAs,p}\left[gC\cdot L^{-1}\right]}{{DOC}_{p}\left[g\cdot L^{-1}\right]}\right\}\times 100$$

where $c_{SCFAs,p}$ represents the mass concentrations of SCFAs (as carbon equivalents) in the permeate, and ${DOC}_{p}$ is the concentration of dissolved organic carbon (DOC) in the permeate.

## 3. Results and Discussions

In this study, there are three major parts: (1) optimization of flocculation at lab scale to produce a low solids filtrate (discussed in Section 3.1.1), and optimization of microfiltration at pilot scale to determine an optimum method to ensure long-term filtration (discussed in Section 3.1.2); (2) semi-continuous operation of treatment chain comprising pilot-scale dark fermentation (Section 3.2.1), chamber filter press (Section 3.2.2), and microfiltration (Section 3.2.2 and Section 3.2.3) under optimized conditions; and (3) evaluation of SCFA recovery out of the treatment chain (Section 3.3).

### 3.1. Optimization of Flocculant Dosages at Lab Scale and Microfiltration at Pilot Scale

#### 3.1.1. Lab-Scale Flocculation of Hydrolyzed Primary Sludge

Initially, a lab-scale study was conducted on hydrolyzed primary sludge or hydrolyzate with total solids (TS) content in the range of approximately 30 to 50 g∙L^−1^ to test the flocculation efficiency of the starch-based flocculant. Experiments were performed to test the dosage requirement, and its influence on the retention of TS, and the attainable effluent quality. Potato-based hydroxypropyl trimethyl ammonium starch (HPAS) was used for the batch tests, as described in Section 2.1.2. Initially, three batch experiments were carried out with a 630 µm sieve (pH 6 to 6.3), and the trend in retained TS on the sieves was monitored. In the first two batch tests (see Table 1), there was a rise in retained TS, which was followed by a breakpoint. The increase in TS suggests the destabilization of the negatively charged colloids, while the further addition of HPAS led to a decline in retained TS due to the excess positive charge from HPAS causing repulsion and the eventual restabilization [7]. In the first two batch tests, the highest value of retained TS was at a similar HPAS dosage (D_HPAS_) of 33 mg_HPAS_∙g_TS_^−1^, after which a breakpoint was observed. The third batch showed no breakpoint, and the TS retained on the sieve increased until a D_HPAS_ of 45 mg_HPAS_∙g_TS_^−1^. This effect was suspected to have been caused by the contents of the hydrolyzate, which was evaluated further in the 100 µm batch tests.

In the 100 µm batch tests (fourth and fifth batch; pH 8.97), there appeared to be a delayed response in the breakpoint in the fourth batch in comparison with the fifth batch (see Table 1). In the fourth batch, the highest retained TS (79 g∙kg^−1^) was achieved at a D_HPAS_ of 39 mg_HPAS_∙g_TS_^−1^, and this dosage was significantly higher than the fifth batch (25 mg_HPAS_∙g_TS_^−1^). Interestingly, the concentration of SCFAs and DOC was higher in the fourth batch (3210 mg_DOC_∙L^−1^; 5119 mg_SCFAs, HAc_∙L^−1^) than in the fifth (2220 mg_DOC_∙L^−1^; 4188 mg_SCFAs, HAc_∙L^−1^). During dark fermentation, complex organic compounds are broken down into simpler molecules, which are then fermented into SCFAs. A large part of the dissolved organic fraction can be in the form of molecules that contain carboxylic acid groups, which can be deprotonated at higher pH-values. In addition to the dissolved components, it is also important to consider the negative charges on microbes [7,17], and a more hydrolyzed sludge can contain more biomass. Overall, this increases the negative charge in the hydrolyzate, and the amount of HPAS required to neutralize the negative charge in the hydrolyzate increases. Therefore, the lack of breakpoint in the third batch could be that the sludge obtained was hydrolyzed more, requiring a higher dosage of HPAS.

Looking into the effect of pH, the flocculation efficiency was compared between the two sieves. TS retained on the sieve was normalized to TS in the hydrolyzate (i.e., ratio of TS retained on the sieve to TS in the hydrolyzate; see Table 1). The average values of the ratio of TS retained on the sieve normalized to TS in the hydrolyzate was 2.2 ± 0.3 for the 630 µm batch tests (pH 6 to 6.3), while it was 2.1 ± 0.2 for the 100 µm batch tests (pH 8.97). It appeared as though an alkaline pH did not have a significant effect on flocculation efficiency (within the tested range), as the filtrate in the fifth batch (pH 8.97) also showed a low total suspended solids (TSS) concentration of 330 mg∙L^−1^ (see photograph of filtrate in Table 1 for fifth batch at a D_HPAS_ of 30 mg_HPAS_∙g_TS_^−1^). In line with this finding, it was shown that a positive charge density for cellulose-based flocculant could be held relatively stable between pH 7 and 9, while a significant decline was observed only when the pH value was increased to 10 [24].

Evaluating the filtrate in the fourth and fifth batch tests (100 µm), sludge conditioning was of absolute necessity, as R_TS_ was very low without conditioning. In both batch tests (100 µm) without conditioning, R_TS_ was less than 60%, while optimum dosages could remove more than 75% of TS. It is necessary to maintain a D_HPAS_ within an optimum range to avoid having high TSS concentration in the filtrate, which can significantly increase a cake layer formation on the microfiltration membrane [25], and lead to a higher retention of SCFAs due to adsorption on particles. Since dry matter content in the hydrolyzate can change on a daily basis, the optimum dosage cannot be always maintained as it takes at least 48 h to determine the dry matter content. However, based on the range of total solids in the hydrolyzate in this study, a filtrate with a low TSS could be achieved within the tested range of flocculant dosage.

#### 3.1.2. Optimization of Pilot-Scale Microfiltration with Filtrate of Chamber Filter Press

Following the optimum dosage that was determined at lab scale, dewatering was performed using a pilot-scale chamber filter press at a dosage of 28 mg_HPAS_∙g_TS_^−1^ to produce a filtrate that could be then used for optimizing microfiltration. The dewatering procedure produced a filtrate with low TS and TSS concentrations, as shown in Table 2. Since the aim was to optimize microfiltration based on the produced filtrate, this particular pilot-scale dewatering experiment was not assessed in detail. However, the analysis of retention parameters for the filter press was performed in detail during the semi-continuous operation in Section 3.2.2. In any case, the objective was to optimize microfiltration with the filtrate to ensure long-term operation without fouling, which could then be used for semi-continuous filtration. Concentrates and permeates were recycled back into the feed solution (recirculation condition, as shown in Figure 1) to maintain a consistent feed composition. All experiments were performed at cross-flow velocity averaging at 3 ± 0.2 m∙s^−1^. The details of the experiment and the composition of the filtrate can be found in Table 2.

**Figure 2:**
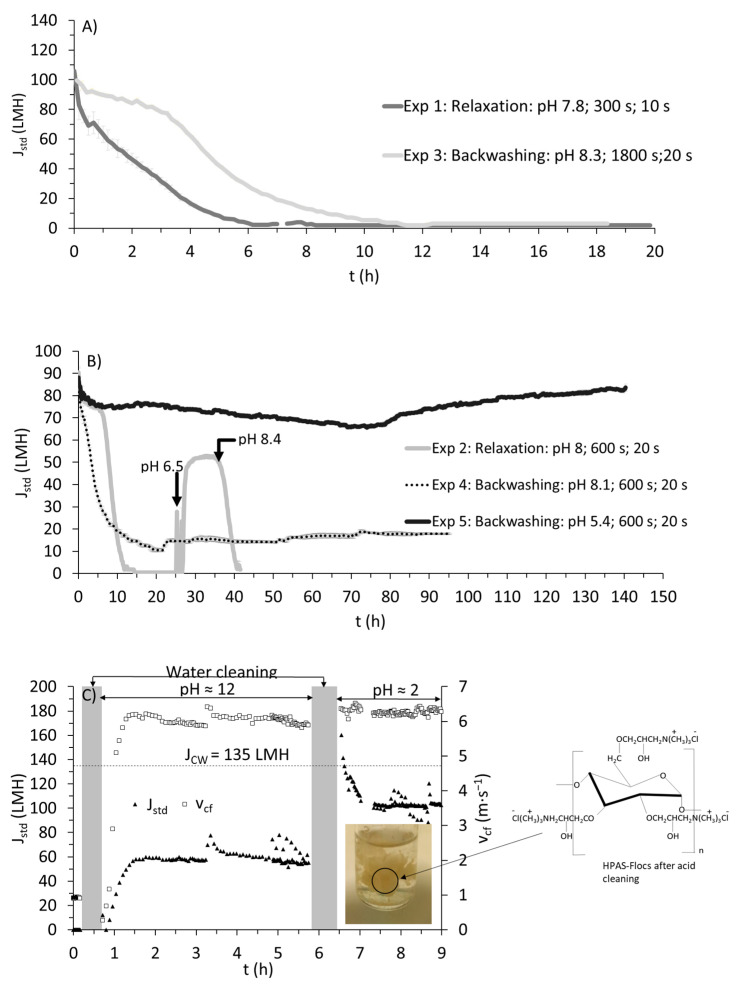
(**A**) Variation of standardized permeate flux (J_std_) with time for A) Experiments 1 (relaxation) and 3 (backwashing), (**B**) Experiments 2 (relaxation), 4 (backwashing), and 5 (backwashing), and (**C**) chemical cleaning (the dashed line indicates clean water flux (J_CW_)). The photograph shows the desorbed flocs of hydroxypropyl trimethyl ammonium starch after removing the waste stream from the microfiltration system. Note: The data for flux are obtained every 5 s. Average and standard deviations are made at 10 min intervals.

Experiment 1 was performed with relaxation cycles, wherein the pressure would be released every 300 s for 10 s, allowing the cross flow to scour the membrane surface. Interestingly, a loss of standardized flux (i.e., J_std_) occurred in the first 6 h (see Figure 2a) of filtration despite the low total suspended solids (TSS) concentration and the high cross-flow velocity used. It is important to consider the nature of the foulants, and not only the concentration. Hydroxypropyl trimethyl ammonium starch (HPAS) can foul the membrane via electrostatic interaction, as ceramic membranes have an iso-electric point (IEP). For α-Al_2_O_3_, IEP was found to be in the range of 8 to 9 [13]. Since the pH value is in the range of IEP (pH 7.8), unbound cationic HPAS or particles bound to HPAS (referred to as HPAS-flocs) are hypothesized to have caused this rapid fouling via electrostatic attraction (with negatively charged α-Al_2_O_3_). Such a phenomenon was also reported for polymeric membranes [26], and the authors identified that pore blocking was the major mechanism by organic flocculants.

This phenomenon can be further supported by a cleaning procedure employed after complete fouling in Experiment 1 (see Figure 2c). Initially, chemical cleaning was performed with an alkaline solution, which partially restored clean water flux J_CW_ (40 to 50% compared with actual J_CW_; see Figure 2c). However, with acid cleaning, around 80 to 90% of J_CW_ could be recovered. The drained solution (after acid cleaning) showed visible flocs, resembling flocs formed during flocculation before dewatering (as shown in the photograph in Figure 2c). Acid cleaning is generally performed to remove inorganic fouling via solubilization. Organic fouling is more predominant in microfiltration with wastewater [27]; therefore, the hydroxide ions, along with chelating agents, should have completely removed such a fouling either via saponification, chelation, hydrolysis, and/or solubilization. But the higher recovery of J_CW_ with acid cleaning implies a drastic shift towards a more positively charged membrane which should have repelled HPAS back to the bulk solution.

It should be noted that the cleaning solutions usually contain other compounds that contribute towards chelation or emulsification, but information on the exact composition of such solutions is not known. To differentiate the effect of membrane surface charge (due to the acid solution’s contact with the membrane) from possible chemical agents that could have desorbed HPAS, Experiment 2 was performed under similar physical cleaning conditions (as Experiment 1 with minor variations, see Table 2), and, similar to Experiment 1, the membrane was susceptible to immediate fouling. A decline in J_std_ occurred in the first 12 h. At around 25 h, the pH in the feed solution was reduced from pH 8 to pH 6.5 with HCl. The sudden reduction in pH and the coincidental increase in J_std_ (see Figure 2b) support the idea that charge interaction plays a significant role in the attraction/repulsion of unbound HPAS or HPAS-flocs. Also, when the pH value reached 8.4, there was a decrease in J_std_ (as shown in Figure 2b), supposedly indicating how a more negatively charged α-Al_2_O_3_ could facilitate more attraction of unbound HPAS or HPAS-flocs.

Chemical cleaning was again performed with respect to the procedure described in Figure 2c to recover J_CW_. To improve upon optimization, backwash cycles were introduced as an alternative cleaning procedure in Experiment 3 (see Table 2). During backwashing, the permeate flow would be reversed to remove any fouling in the pores and the membrane surface [28]. In Experiment 3, backwashing was performed every 1800 s for 20 s. Such a frequency did not have any effect on the stability of J_std_, as it saw a rapid decline at around 10 h (Figure 2a). However, when the frequency of backwashing was increased to every 600 s for 20 s (Exp 4; Figure 2b), a rather stable J_std_ of around 20 LMH was observed after an initial decline in J_std_. This hints at how an inevitable interaction of HPAS with the membrane can severely cause fouling despite the low TSS concentration. In any case, the initial interaction of HPAS was unavoidable, but, with a higher frequency, fouling and pore blocking could be reduced, allowing for long-term filtration. In comparison, using the same membrane (v_cf_ = 3 m∙s^−1^, pressure = 2.2 bars, without physical cleaning) to treat swine manure, a steady state flux of 20 LMH for a 3-day period was attained under recirculation conditions [15]. However, the TSS concentration in the study was around 5 g∙L^−1^, and was found to have contributed majorly towards membrane fouling. A similar range of flux was achieved with polymeric microfiltration membrane (pore size = 0.3 µm) for filtering activated sludge with TSS of 5800 mg∙L^−1^ [25]. The presence of particles was found to be the major cause of fouling resistance in their study.

Nevertheless, to further support the phenomenon of HPAS fouling and improve upon J_std_, Experiment 5 was performed with the filtrate at a rather low pH (pH 5.4), with the optimized backwashing conditions from Experiment 4 (see Table 2 for experimental conditions). The experiment was carried out for 168 h, and maintained around 70 to 80 LMH, and demonstrated no signs of decline in J_std_. This clearly shows that HPAS was the major cause for fouling, and the rapid decline in J_std_ at the start of filtration was caused by HPAS or HPAS-flocs. With a low pH, the initial electrostatic interaction of HPAS with the membrane could be significantly reduced, allowing for a high J_std_ to be maintained long term. In addition, the increased backwashing frequency could significantly prevent pore blocking. This option could still be considered for continuous filtration, as the amount of volume that can be filtered is significantly higher. After a filtration duration of 96 h, approximately 6840 L per m^2^ of effective membrane area could be filtered with conditions from Experiment 5, which was around 3.9 times higher than with Experiment 4 (1770 L∙m_eff_^−2^) with backwashing.

### 3.2. Cascade of Dark Fermentation and Two-Step Membrane Separation

#### 3.2.1. Dark Fermentation at Different Organic Loading Rates

**Figure 3:**
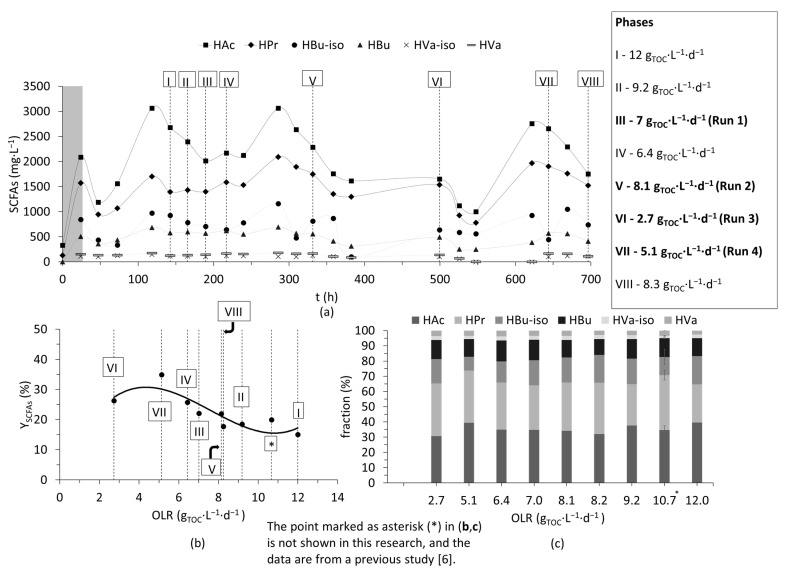
(**a**) Variation in concentration of short-chain fatty acids (SCFAs) in the hydrolyzate with time. The roman numerals marked in (**a**) denote the different phases. Arbitrary days (denoted as runs) were chosen for membrane separation (marked in bold in the text box in (**a**)). (**b**) Yields of SCFAs (YSCFAs) based on the organic loading rate (OLR). (**c**) Fraction (%) shows the percentage of individual SCFAs accounting for the total SCFAs. The point marked as asterisk (*) in (**b**,**c**) is not shown in this research, and the data are from a previous study [6].

Following the optimization of microfiltration, a cascade experiment combining dark fermentation and two-step membrane filtration was performed to evaluate SCFA recovery from the solids stream. Firstly, dark fermentation was performed for around 30 days, and, during this period, SCFA production varied significantly due to changes in OLR. As key parameters like pH (pH 6.9 ± 0.1), temperature (T = 32 °C), and HRT (36 h) were already optimized based on the previous study [6], the changes in SCFA concentration could be monitored for variations in OLR. Arbitrary days (shown as phases I to VIII in Figure 3a) were chosen to evaluate the effect of OLR on yields of SCFAs ($Y_{SCFAS}$). All phases were carried out at optimized conditions, excluding phase VI, which had an HRT of 72 h (other parameters like pH and temperature were similar). The change in HRT was due to a shortage of required sludge volume to maintain 1.5-day HRT.

Irrespective of the different OLRs, the percentage of individual SCFAs accounting for total SCFAs was slightly similar (see Figure 3c). However, when evaluating $Y_{SCFAS}$, a stability over a wide range (between 7 and 12 g_TOC_∙L^−1^∙d^−1^) was observed (see Figure 3b) but increased below 7 g_TOC_∙L^−1^∙d^−1^. The highest yields were observed at phases IV (6.4 g_TOC_∙L^−1^∙d^−1^), VI (2.7 g_TOC_∙L^−1^∙d^−1^), and VII (5.1 g_TOC_∙L^−1^∙d^−1^) (see Figure 3b). At lower OLRs, $Y_{SCFAS}$ could be improved by around 15 to 50%. OLR is a function of both HRT and concentration of the substrate (here TOC). As mentioned earlier, HRT at phase VI was 72 h, and the higher $Y_{SCFAS}$ at this phase VI could be attributed to longer residence times, but it is important to mention that a plateau of $Y_{SCFAS}$ was achieved at around HRT of 48 h in the previous study [6], and $Y_{SCFAS}$ (here in phase VI) was also lower than or close to the yields observed at phases IV and VII (both of which had HRT of 36 h). Therefore, the effect of lower OLR was suspected to have caused this increase at phase VI.

In phases IV, VI and VII, sludge was relatively diluted, with TOC concentration being at least 25% lower compared with other phases. Although, higher OLRs can enhance fermentation, it appears as though maintaining a relatively low/optimum OLR can increase the overall yields of SCFAs [29]. Similarly, it was also shown that a slightly diluted substrate or low sludge loading periods improved SCFA production significantly, which was mainly attributed to a reduced inhibitory effect of the concentrated substrate (i.e., organic carbon) on the biomass, and better mixing and uptake of the substrate by the biomass during low sludge loading periods [30]. Similar results were also reported [31], where the authors showed an optimum dilution for maximizing SCFA yields, beyond which a decrease was observed, and the reason was attributed to reduced inhibitory effects at optimum concentrations.

#### 3.2.2. Pilot-Scale Dewatering and Microfiltration: Evaluation of Retention

The hydrolyzate obtained after dark fermentation (from the four runs that are marked in Figure 3a) was then treated using a chamber filter press to reduce the solids load on microfiltration. A total of four dewatering runs were carried out. Pea-based hydroxypropyl trimethyl ammonium starch (HPAS) was used here, and the dosage (D_HPAS_) was increased from run 1 to run 4 to monitor the effects of solids removal.

Despite a lower D_HPAS_ in comparison with the batch tests, a high total solids retention (R_TS_) was observed (Table 3). This can be explained by the HPAS used for pilot-scale dewatering, which was pea-based (as mentioned in Section 2.1.2). The pea-based flocculant had a higher degree of substitution (DS), meaning that the number of OH groups replaced in starch by a quaternary ammonium salt was significantly higher compared with the potato-based HPAS (used for lab scale). The latter had a DS value less than 1.6 (DS < 55%; exact value is unknown), while that of the former was 3 (theoretically 100%). A higher DS could reduce the dosage requirements, and it was also found that the flocculation efficiency of starch-based flocculants with a higher DS value was higher [7]. However, the authors also noted that there was an increased risk of faster stabilization due to the higher charge density, which was suspected to have occurred in run 4, as there was a breakpoint in retention. R_TS_ could reach an average of 67 until run 3, past which there was a significant reduction in R_TS_ to 55%. In addition, the ratio of the concentration of total suspended solids (TSS) in the filtrate to the concentration of TS in the hydrolyzate (expressed as mg∙g^−1^; see Table 3) could be kept to a minimum in the filtrate (6 mg∙g^−1^) at a D_HPAS_ of 19 mg_HPAS_∙g_TS_^−1^, after which there was an increase. In retrospect, to obtain such a low value (9 mg∙g^−1^), a D_HPAS_ of 30 mg_HPAS_∙g_TS_^−1^ was necessary for the fifth batch (100 µm) in Section 3.1.1. Using the filter press, a volume recovery (V_rec_) of 85% can be achieved.

The filtrate of the chamber filter press from the four runs was then treated with ceramic microfiltration to achieve a particle-free SCFA permeate. Using microfiltration, complete TSS removal could be achieved (R_TSS_ = 100%), and a V_rec_ value (after microfiltration) of 70% could be reached. R_TS_ for microfiltration was in the range of 20% for runs 1, 2, and 4, while it was less than 10% in run 3 (see Table 3). In contrast, a retention of around 80% after 3 days of filtration was found in a study [15], and the authors assumed a significant portion of dissolved organic compounds adsorbed to the suspended solids contributed to such high retention values. This further signifies the importance of having a filter press to reduce the suspended solids load on microfiltration, and eventually prevent the loss of SCFAs. The removal of particles after microfiltration also led to a high permeate quality, as evidenced by the high ratio of SCFAs (as carbon equivalents) to DOC (represented by f_DOC_ in Table 3), and the average values of f_DOC_ from the four runs was around 89%.

In any case, dewatering and microfiltration operate under the mechanism of size sieving, and the retention of low molecular-weight molecules, like SCFAs, should be negligible. However, the retention of SCFAs (R_SCFAs_) has shown to occur in the presence of high concentrations of suspended solids. The co-retention of SCFAs along with TSS has been reported by other researchers for ceramic (0.2 µm) and polymeric membranes (0.1 to 8 µm), and have found to be in the range of 15 to 20% [15,16]. Similarly, large macromolecules like proteins and polysaccharides were reported to be retained by more than 30% by a 0.1 µm ceramic membrane [32]. Also, the narrowing of pores due to adsorption of foulants and cake layer formation are phenomena that lead to the notable rejection of dissolved compounds (in the range of 20%) in the presence of high concentrations of TSS [25]. The filter press lined with polyester membrane showed an R_SCFAs_-value in the range of 1 to 8% (supplementary Figure S1 and Table 3), despite the relatively larger mesh size of 100 µm. The SCFAs produced during dark fermentation had pKa values in the range of 4.7 and 4.9 (at 25 °C), and existed predominantly in the dissociated or deprotonated forms at a pH value of 7 (which was used in dark fermentation). It is important to consider the electrostatic interaction between the cationic HPAS and the carboxylate ion (after dissociation) in SCFAs, which would have led to this retention, and it was observed that the retention of SCFAs increased with the increasing dosage of HPAS (see supplementary Figure S1). In addition to HPAS’s electrostatic interaction, there was a significant concentration of TS in the hydrolyzate, and adsorption to the solids was also suspected to have been a factor that could have contributed to this low retention. However, there was no discernible pattern that can support the theory of adsorption to solids (see Table 3). In this study, R_SCFAs_ was found to be relatively low for the ceramic microfiltration membrane (0.2 µm). The average R_SCFAs_ was around 5% for short- and long-term experiments conducted in Section 3.1.2 and also in the four runs in semi-continuous microfiltration (see supplementary Figure S1 and Table 3). Based on observations from the studies mentioned above, the low TSS concentration in the filtrate (i.e., feed for microfiltration) could be a major factor preventing the significant retention of SCFAs.

#### 3.2.3. Performance of Microfiltration Membrane During Semi-Continuous Operation

**Figure 4:**
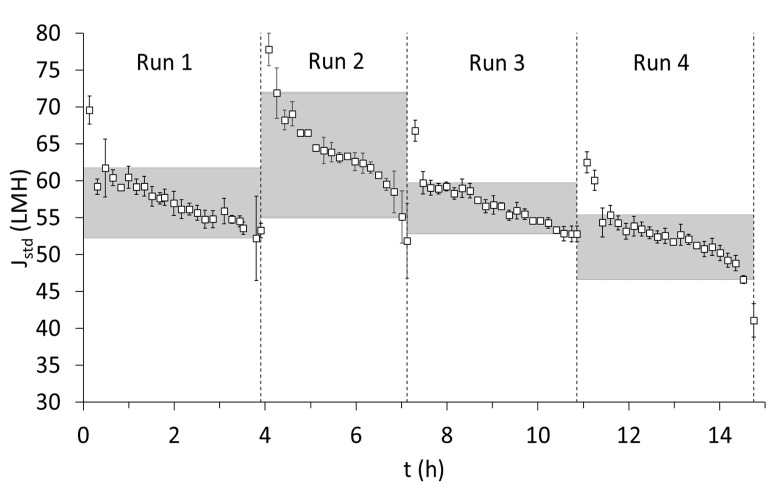
Standardized flux (J_std_) for the 4 runs. Hydrolyzate from the 4 days mentioned in Figure 3 after pre-treatment with filter press was used for microfiltration. Conditions: pH 7, see Table 3 for parameters of the feed solution (filtrate). Note: The data for flux are obtained every 5 s. Average and standard deviations are made at a 10 min interval.

Semi-continuous microfiltration was performed with backwashes every 600 s for 20 s based on the stable results obtained from the recirculation condition in Section 3.1.2. Average cross-flow velocity during the four runs was 3 ± 0.3 m∙s^−1^. It is important to remember that, similar to the filter press, semi-continuous filtration was performed, but no interim chemical cleaning was performed between the four runs. The membrane underwent only cleaning with clean water between each run. The duration of microfiltration was between 3 and 4 h for all four runs. In all four runs, after a steep decline in standardized flux (J_std_), there was a stable phase that was represented by the upper and lower limits of J_std_ during the filtration period of each run (see the shaded region in Figure 4). In runs 1,2, and 3, the stability of J_std_ was maintained during the course of filtration, while, in run 4, there was a notable deviation, which could very well indicate the onset of irreversible fouling (around 40 LMH was reached, which was below the lower limit of the shaded region). Further examination into the trend of J_std_ during short-term filtration showed that J_std_ was much higher for the experimented duration compared with the long-term experiment performed under similar physical cleaning conditions (Experiment 4 in Section 3.1.2; see Figure 2b). In Experiment 4, the range of 50 to 60 LMH could only be maintained for a period of 3 h, which was almost close to the duration of filtration of one run. One reason could be attributed to the reduced dosage of hydroxypropyl trimethyl ammonium starch, while another possible explanation would be the inclusion of short intermittent cleaning with clean water, which could potentially sustain J_std_ for longer durations. Nevertheless, by the end of run 4, a total of around 900 L∙m_eff, area_^−2^ of filtrate was treated; thus, this is the amount of filtrate after which chemical cleaning must be performed to restore the flux in accordance with the procedure described in Section 3.1.2 and Figure 2c.

### 3.3. Evaluation of the SCFA Load Recovered out of the Cascade of Dark Fermentation and Two-Step Membrane Separation

**Figure 5:**
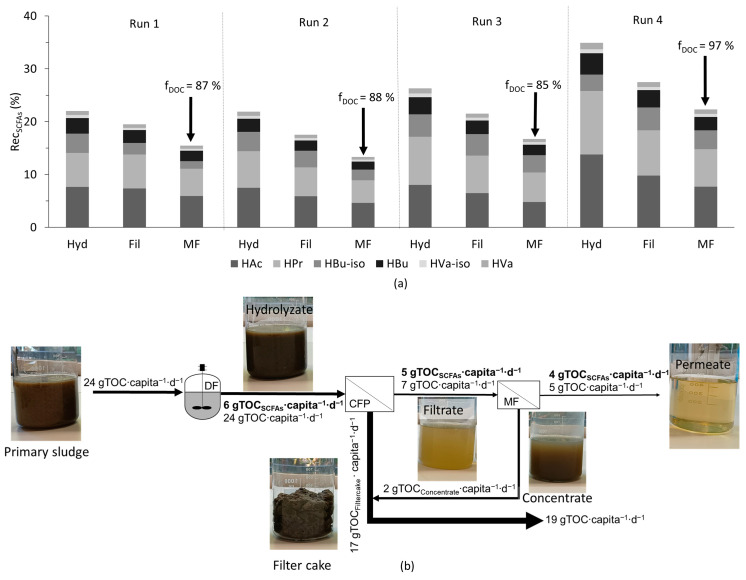
(**a**) Recovery of SCFAs (Rec_SCFAs_) after each step, including volume recovery of filtration, and (**b**) organic carbon load in a biorefinery expressed as TOC. Excess sludge was included in the load and, for the calculation, the yield from the treatment chain with primary sludge was used. Note: DF - Dark fermentation, CFP – Chamber filter-press, MF – Microfiltration.

Although with microfiltration a particle-free permeate could be produced with permeate quality (f_DOC_) of 85 to 97%, there was a drawback in terms of the decline in the overall recovery of SCFAs (Rec_SCFAs_), which can be attributed to volume recovery (V_rec_) (see Figure 5a). It is important to mention that the 70% V_rec_ achieved with microfiltration was a system limitation, and the recovery of SCFAs (Rec_SCFAs_) could be increased by having a higher V_rec_, provided there was not a high significant retention of SCFAs. Nevertheless, the overall value of Rec_SCFAs_ after microfiltration was around 15%. However, with the observed increase in yields at low OLRs in dark fermentation, the Rec_SCFAs_ could be potentially increased by at least 20%. From both primary and excess sludge, around 24 g_TOC_∙capita^−1^∙d^−1^ was generated. Based on the yields observed in this study after microfiltration (with primary sludge), around 4 gC_SCFAs_∙capita^−1^∙d^−1^ could be recovered from the solids stream (primary and excess sludge) of municipal WWTP (see Figure 5b).

**Figure 6:**
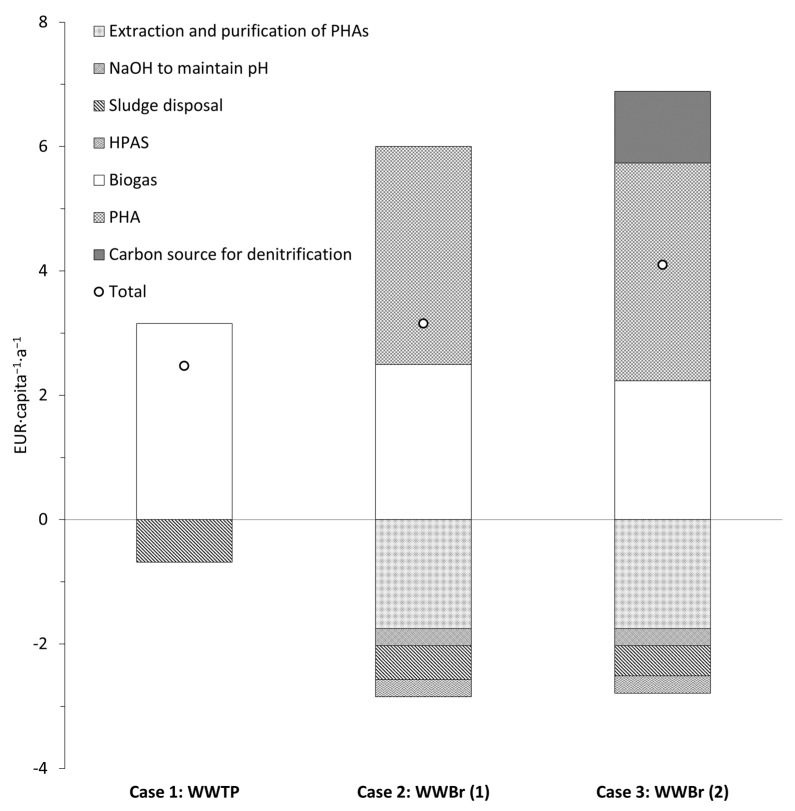
Revenue–expenditure analysis for wastewater treatment plant and wastewater biorefinery.

To understand the feasibility of the particle-free permeate and its significance in a wastewater biorefinery (WWBr), a simple revenue–expenditure analysis was performed in terms of per capita and per annum (a) based on two cases (Figure 6). In case 1, a typical wastewater treatment plant (WWTP) was assessed, with biogas as the major source of revenue, while, in case 2, PHA recovery with SCFAs obtained as a particle-free permeate after microfiltration in a WWBr was evaluated.

The organic carbon load was 24 g_TOC_∙capita^−1^∙d^−1^ produced as primary and excess sludge. For case 1, the revenue generated was based on the conversion of organic carbon to biogas. The amount of revenue generated based on the electrical energy obtained (from methane) through combined heat and power was 0.36 EUR∙kg_TOC_^−1^ [2]. Therefore, based on the TOC load (24 g_TOC_∙capita^−1^∙d^−1^), this was equivalent to a revenue of 3.15 EUR∙capita^−1^∙a^−1^, while the cost associated with sludge disposal was around EUR 100 per ton of TS [33]. Based on the solids load generated, the sludge disposal costs amounted to 0.68 EUR∙capita^−1^∙a^−1^.

For case 2, the revenue generated from biogas was reduced, as a sizeable portion of the organic carbon was diverted to the recovery of SCFAs (as shown in this study). Firstly, for WWBr, two scenarios can be considered for biogas production. For scenario 1 in case 2 (case 2: WWBr (1)), both the filter cake (from the chamber filter press) and the concentrate of microfiltration, which amounted to a load of 19 g_TOC_∙capita^−1^∙d^−1^, was used for biogas production, generating a revenue of 2.5 EUR∙capita^−1^∙a^−1^ (0.36 EUR∙kg_TOC_^−1^ [2]). This revenue reduced to 2.2 EUR∙capita^−1^∙a^−1^ when only the filter cake was used (17 g_TOC_∙capita^−1^∙d^−1^), while the concentrate of microfiltration was used for process optimization (case 2: WWBr (2)). The concentrate of microfiltration had a notable load of SCFAs (around 2 g_TOC_∙capita^−1^∙d^−1^; see Figure 5b), which could be used for biological nutrient removal. Interestingly, the SCFA mixture obtained from the hydrolyzed primary sludge (after centrifugation) improved the rates of denitrification in comparison with HAc [34]. In the case of pre-anoxic treatment in a municipal WWTP, there would be periods of low organic carbon load in the influent, and this could be met with the SCFA load in the concentrate of microfiltration, which could reduce the cost of external HAc (0.63 EUR∙kg_HAc_^−1^ Business Analytik (2024)). For PHA calculation in case 2, an average yield of 0.33 g C_PHA_/gC_SCFAs_ was chosen [35] and an overall revenue of 3.5 EUR∙capita^−1^∙a^−1^ could be generated (at an average selling price of 4 EUR∙kg_PHA_^−1^ [36]). However, around 50 % of the revenue generated by PHAs was offset by the extraction and purification costs [2]. Other major costs included the dosage requirements of NaOH and starch for dewatering, which were 0.14 g_NaOH_∙g_TOC_^−1^ (0.22 EUR∙kg^−1^; from Business Analytik (2024)) and 0.02 g_HPAS_∙g_TOC_^−1^ (1.6 EUR∙kg_HPAS_^−1^; HKF CleanTech AG, Rotkreuz, Switzerland, respectively, which accounted for 0.55 EUR∙capita^−1^∙a^−1^. The energy demand for microfiltration was low, and did not have a notable effect on the cost and is not included). In conclusion, the revenue generated in a WWBr can be increased by an average of 50% based on case 2 (see Figure 6).

## 4. Conclusions

Implementing dark fermentation in combination with filter press and microfiltration is a viable option to produce a particle-free short-chain fatty acid (SCFA) permeate from the solids stream of municipal wastewater.

(1) Dark fermentation stands as a crucial step and the yields of SCFAs can be potentially enhanced by at least 20% by maintaining lower organic loading rates (2–5 g_TOC_∙L^−1^∙d^−1^).
(2) Filter press (mesh size: 100 µm) coupled with hydroxypropyl trimethyl ammonium starch (HPAS) is an effective pre-treatment stage and can remove more than 60% of the solids and produce a filtrate with low suspended solids concentration.
(3) HPAS contributes significantly to fouling of microfiltration, but fouling can be mitigated by increased backwashing frequency, and also by reducing solution pH below membrane iso-electric point.
(4) Approximately 4 gC_SCFAs_∙capita^−1^∙d^−1^ can be recovered in a particle-free permeate from the sludge stream of a wastewater biorefinery.

## Figures and Tables

**Table 1:** Gravity driven batch tests with different sieve sizes to test flocculation efficiency. The photographs are representative of the filtrate from the fifth batch test with 100 µm sieve.

Sieve Size	pH	TS in Hydrolyzate	Batch Number										
600 µm	pH 6.3	38 g∙L^−1^	First batch	D_HPAS_ (mg_HPAS_∙g_TS_^−1^)	0	13	20	25	33	38	51	50	
				TS retained on sieve (g∙kg^−1^)	-	77	83	87	91	90	86	86	
				$\frac{TS\;retained\;on\;sieve\;(g\cdot {kg}^{-1})}{TS\;in\;hydrolyzate\;(g\cdot {kg}^{-1})}$(−)	-	2	2.2	2.3	2.4	2.4	2.3	2.3	
	pH 6	45 g∙L^−1^	Second batch	D_HPAS_ (mg_HPAS_∙g_TS_^−1^)	0	20	22	25	27	30	33	39	49
				TS retained on sieve (g∙kg^−1^)	-	79	86	88	82	82	97	89	83
				$\frac{TS\;retained\;on\;sieve\;(g\cdot {kg}^{-1})}{TS\;in\;hydrolyzate\;(g\cdot {kg}^{-1})}$(−)	-	1.8	1.9	2	1.8	1.8	2.2	2	1.8
		36 g∙L^−1^	Third batch	D_HPAS_ (mg_HPAS_∙g_TS_^−1^)	0	13	20	27	34	39	45		
				TS retained on sieve (g∙kg^−1^)	79	82	82	89	89	92	93		
				$\frac{TS\;retained\;on\;sieve\;(g\cdot {kg}^{-1})}{TS\;in\;hydrolyzate\;(g\cdot {kg}^{-1})}$(−)	2.2	2.3	2.3	2.5	2.5	2.6	2.6		
100 µm	pH 8.7	33 g∙L^−1^	Fourth batch	D_HPAS_ (mg_HPAS_∙g_TS_^−1^)	0	18	27	30	39	41			
				TS retained on sieve (g∙kg^−1^)	63	65	67	70	79	78			
				$\frac{TS\;retained\;on\;sieve\;(g\cdot {kg}^{-1})}{TS\;in\;hydrolyzate\;(g\cdot {kg}^{-1})}$(−)	1.9	2	2	2.1	2.4	2.4			
				TS in the filtrate (mg∙L^−1^)	14,197	9519	9439	8329	8075	8378			
				R_TS_ (%)	57	71	72	75	76	75			
		35 g∙L^−1^	Fifth batch	D_HPAS_ (mg_HPAS_∙g_TS_^−1^)	0	18	25	30	36	39			
				TS retained on sieve (g∙kg^−1^)	65	73	77	72	65	66			
				$\frac{TS\;retained\;on\;sieve\;(g\cdot {kg}^{-1})}{TS\;in\;hydrolyzate\;(g\cdot {kg}^{-1})}$(−)	1.9	2.1	2.2	2.1	1.9	1.9			
				TS in the filtrate (mg∙L^−1^)	14,754	10,372	8929	8288	7982	8086			
				R_TS_ (%)	58	71	75	76	77	77			
				TSS in the filtrate (mg∙L^−1^)	-	-	630	330	630	750			
				$\frac{TSS\;in\;the\;filtrate\;(mg\cdot L^{-1})}{TS\;in\;hydrolyzate\;(g\cdot L^{-1})}$ (mg∙g^−1^)	-	-	18	9	18	21			
				Visual observation of filtrate	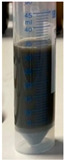	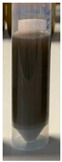	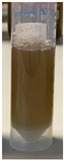	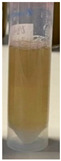	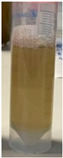	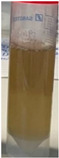			

**Table 2:** Description of experiments performed with cross-flow ceramic tubular microfiltration (VSS ≈ TSS).

Experiment	Type of Cleaning	Conditions	Frequency of Physical Cleaning	Duration of Physical Cleaning	Initial pH	TS (mg∙L^−1^)	TSS (mg∙L^−1^)	TOC (mg∙L^−1^)	DOC (mg∙L^−1^)
1	Physical	Relaxation	300 s	10 s	7.8	6262 ± 619	430 ± 94	2326 ± 255	1508 ± 304
2	Physical/ chemical	Relaxation	600 s	20 s	8				
3	Physical	Backwashing	1800 s		8.3				
4	Physical	Backwashing	600 s		8.1				
5	Physical/chemical	Backwashing			5.4 ^a^				

^a^ The pH-value was reduced from 8 to 5.4 with HCl.

**Table 3:** Parameters of the inflow and outflow of chamber filter press and microfiltration.

Runs	Hydrolyzate Obtained from Dark Fermentation		Dosage (D_HPAS_)	Filtrate After Dewatering of Hydrolyzate Using Chamber Filter Press				Permeate After Microfiltration of Filtrate		
	TS	SCFAs		TSS	$\frac{TSS\;in\;filtrate\;(mg\cdot L^{-1})}{TS\;in\;hydrolyzate\;(g\cdot L^{-1})}$	R_TS_	R_SCFAs_	R_TS_	R_SCFAs_	f_DOC_
	g∙L^−1^	mg∙L^−1^	mg_HPAS_∙g_TS_^−1^	mg∙L^−1^	mg_TSS_∙g_TS_^−1^	%		%		
1	26	4926	6	1300	50	65	1	21	4 ± 2	87
2	30	5686	10	1281	43	68	6	21	4 ± 3	88
3	21	4551	19	115	6	67	4	6	6 ^a^	85
4	19	5831	25	348	18	55	8	21	3 ± 1	97

^a^ The standard deviation was not included as the coefficient of variation was very low (less than 2%). Note: Total solids (TS) and short-chain fatty acids (SCFAs) shown in Table 3 were measured in duplicates in the hydrolyzate before dewatering and in the filtrate after dewatering for calculation of retention parameters. For microfiltration, TS was measured in duplicate in the feed before filtration and at the end of the filtration for retention calculation. For calculation of SCFA retention in microfiltration, SCFAs were measured once in duplicate in the feed, once in permeate (duplicate) when half the volume recovery was achieved, and once again in the permeate (duplicate) when the filtration was performed where a maximum volume recovery could be achieved. The measurements were then averaged.

## Data Availability

Data will be made available at request.

## Supplementary Materials

The following supporting information can be downloaded at: https://www.mdpi.com/article/10.3390/membranes15010022/s1, Figure S1: (A) Retention of short-chained fatty acids (SCFAs) R_SCFAs_ for a pilot-scale chamber filter press lined with a polyester membrane (pore size = 100 µm) at different dosages of hydroxypropyl trimethyl ammonium starch (HPAS), and (B) R_SCFAs_ for ceramic microfiltration membrane (pore size = 0.2 µm) during short- and long-term microfiltration experiments 1 to 5 and semi-continuous microfiltration runs 1 to 4. Note: In Figure S1B, for experiments 1 to 5, samples were taken on a daily basis and, from all the values combined, a histogram was generated. For runs 1 to 4, 2 samples for each run were combined and then a histogram was generated. Figure S2: Relationship between permeate flow rate and temperature to correct permeate flux for microfiltration.
