# Supplementary material for: Separation of Short-Chain Fatty Acids from Primary Sludge into a Particle-Free Permeate by Coupling Chamber Filter-Press and Cross-Flow Microfiltration: Optimization, Semi-Continuous Operation, and Evaluation
Source: Membranes (Basel). 2025 Jan 11;15(1):22. doi: 10.3390/membranes15010022 (PMC11766653; doi:10.3390/membranes15010022)
Supplement: Supplementary file 1 [file membranes-15-00022-s001.zip › membranes-3394837-supplementary.pdf]

Type of the Paper (supplementary information)

Separation of short-chain fatty acids from primary sludge into a particle-free permeate by coupling chamber filter-press and cross-flow microfiltration: Optimization, semi-continuous operation and evaluation

Nikhil Shylaja Prakash[1,*], Peter Maurer [2], Harald Horn[1,3], Florencia Saravia[1] and Andrea Hille-Reichel[1,3,*]

[1] DVGW-Research Center at the Engler-Bunte-Institut, Water Chemistry and Water Technology, Karlsruhe Institute of Technology, Engler-Bunte-Ring 9, Karlsruhe 76131, Germany; nikhil.prakash@partner.kit.edu (N.S); harald.horn@kit.edu (HH); saravia@dvgw-ebi.de (FS); andrea.hille-reichel@kit.edu (AH)

[2] University of Stuttgart, Institute for Sanitary Engineering, Water Quality and Solid Waste Management, Sewage Treatment Plant for Research and Education, Bandtäle 1, Stuttgart 70569, Germany; peter.maurer@iswa.uni-stuttgart.de (P.M)

[3] Karlsruhe Institute of Technology, Engler-Bunte-Institut, Water Chemistry and Water Technology, Engler-Bunte-Ring 9, Karlsruhe 76131, Germany

* Correspondence: nikhil.prakash@partner.kit.edu; andrea.hille-reichel@kit.edu

S1. Retention of SCFAs by filter-press and microfiltration

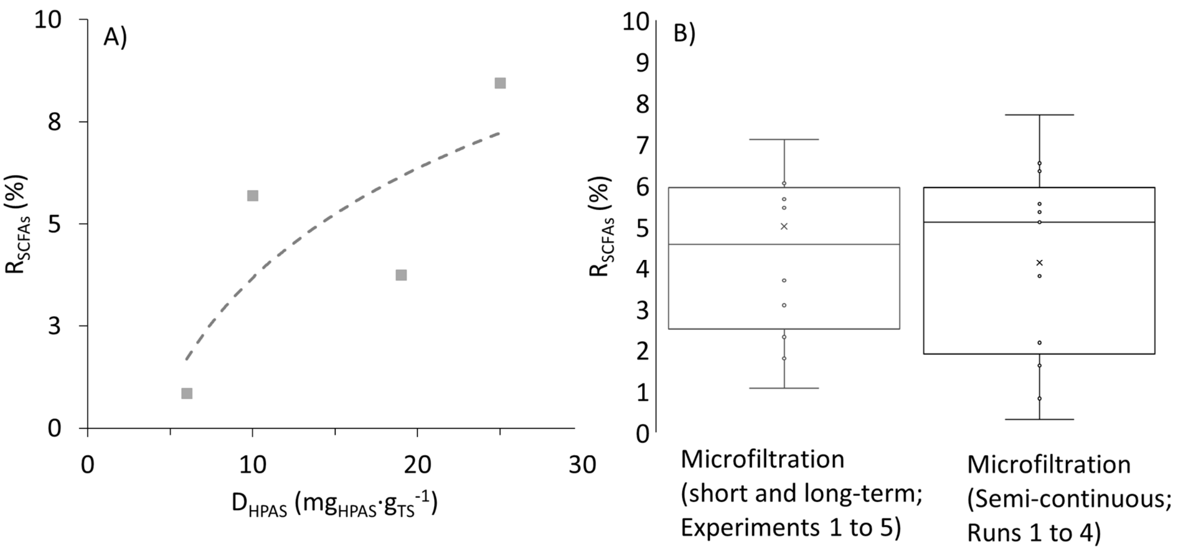

Figure S 1: A) retention of short-chained fatty acids (SCFAs) R_{SCFAs} for a pilot-scale chamber filter-press lined with a polyester membrane (pore size = 100 µm) at different dosages of hydroxypropyl trimenthyl ammonium starch (HPAS), and B) R_{SCFAs} for ceramic microfiltration membrane (pore size = 0.2 µm) during short and long-term microfiltration experiments 1 to 5 and semi-continuous microfiltration runs 1 to 4. Note: in **Figure S1B**, for experiments 1 to 5, samples were taken on a daily basis and from all the values, a histogram is generated. For runs 1 to 4, at least 2 samples for each run were combined and then a histogram was generated.

S2. Relationship between permeate flow rate and temperature

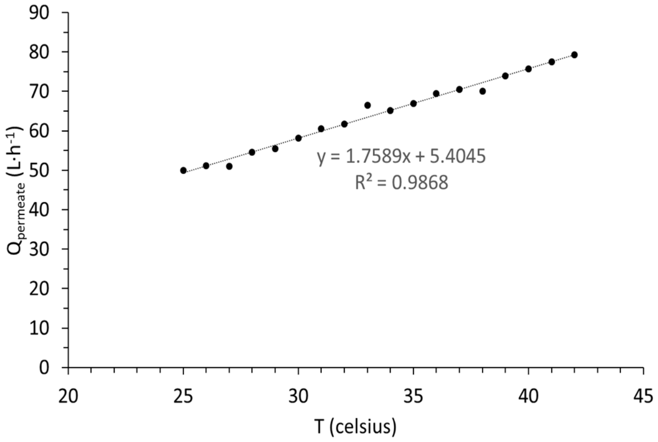

Figure S 2: Relationship between permeate flow rate and temperature to correct permeate flux for microfiltration.
